# Multisensory reading promotion in academic libraries

**DOI:** 10.3389/fpsyg.2022.987180

**Published:** 2023-01-04

**Authors:** Wenyan Yu, Yiping Jiang, Yanqi Wu, Yanxia Cheng

**Affiliations:** Library, Zhejiang University of Technology, Hang Zhou, Zhejiang, China

**Keywords:** multisensory reading, reading promotion, visual, audio, emotional, academic library

## Abstract

To confront college students’ new reading patterns and the continuous decline in academic library borrowing rates, we conducted empirical research on promoting multisensory reading as a way to attract students’ attention, and to stimulate interest in, and promote the practice of, reading through a library program called “Reading Today Listening Everyday” (RTLE) on a library’s WeChat public account. The program involved 48 librarians and 105 students who were recruited into different groups to co-create, edit and release multisensory tweets every workday. Multisensory contents including text-based content, audio-based content and emotional resonance were presented to evoke readers’ visual, audio, and emotional senses to induce more reading practice. Using the Context, Input, Process and Product (CIPP) evaluation method, the multisensory presentation in RTLE program was proven to be effective in promoting library reading with a high number of tweeted page views and an increased borrowing rate for recommended books. In 2020, 269 issues accompanied by 269 audio frequencies garnered 80,268 page views, depending on the caliber of the reading promoter out of the 48 librarians and 52 student anchors behind it. The 484 RTLE-recommended books were borrowed 113 times in 2020, which was a rate 1.46 times higher than in 2019 (77 times). The analysis of the relationship between tweet views and borrowing rates for recommended books indicates that more page views indicate greater reader interest, leading to increased borrowing. From readers’ feedback and comments, the gain afforded by multisensory reading can improve higher-level reading trends such as the number of reading interests, enjoyment, engagement, etc.

## Introduction

The notion that the senses are better conceptualized as interrelated modalities rather than independent channels is supported by many studies, providing evidence for common neural and psychological mechanisms for the processing of multisensory information (Spence et al., 2000; Doyle and Walker, 2002). For example, for a public visiting a museum, simply looking at art works may not be sufficient to interpret them. Sensitivity to additional cues, like background music and audio commentaries, will also guide them to apprehend them. Similarly, one may consider the multisensory attributes of reading. In the process of reading, multisensory reading which involves more readers’ senses should be more interesting and effective. Considering the changing in mass reading habits and strengths of multisensory presentation, the library of Zhejiang University of Technology tries to introduce multisensory mode into reading promotional activities to recommend library collections.

When analyzing changes in college students’ reading patterns, a “sensory turn” could be identified. According to the report of the 18^th^ National Reading Survey in 2021 (The Eighteenth National Reading Survey Report, 2021), the comprehensive reading rate on social media continues to grow steadily, for example, content reading rate on mobile phone for adults was 79.4%, and content hearing rate was 31.6%. Compared with traditional reading, digital reading integrates text, sound, image, and voice, which can bring readers a multisensory and tridimensional reading experience. Intriguingly, and contrary to the increasing reading rate, the book-borrowing rate of many renowned university libraries fell over 50% in recent years (Zheng, 2019), which calls for research on how librarians conduct reading promotional activities to meet students’ growing reading needs and promote the utilization of library collection.

Despite the heartening proliferation of promotional reading programs in academic libraries, many studies reported only their limited success and sustainability (Gauder et al., 2007; Bosman et al., 2008; Fajardo, 2010; Chesnut, 2011; Mackay and Tarulli, 2015; Dali and McNiff, 2020). We view the lack of a differentiated approach to promote reading and the low level of reader engagement in activities as one of the reasons for the limited success and short lifespan of reading initiatives. Unfortunately, there are unexpectedly few studies on this subject. To enrich the literature, We conducted empirical research on reading promotion of multisensory mode as an attempt to attract students’ attention, stimulate reading interest, and promote reading practice, which was implemented in a library program called “Reading Today Listening Everyday” (RTLE) on a library’s WeChat public account, We aimed for our results to provide a new mode to improve library reading promotion with the expectation to meet readers’ needs and increase the utilization rate of library collection. Additionally, the experience of multisensory reading promotion allows librarians to innovate when providing reading services and provide a reference for reading promotion for other libraries, businesses, and policy-planning offices.

## Literature review

While the multisensory aspects of handling books mainly has to do with perception, the multisensory aspects of reading mostly involve the rich mental imagery that is evoked by various forms of presentation (Spence, 2020). Mental imagery is often described as “seeing with the mind’s eyes” and “listening with the mind’s ears,” and it plays an important role in understanding individual cognitive function (Kosslyn, 2005). The multiple sensory aspects of mental imagery that may be at work during reading mainly include visual imagery (Boerma et al., 2016; Brosch, 2018), auditory imagery (Perrone-Bertolotti et al., 2012; Moore and Schwitzgebel, 2018), and the complementary “inner ear” non-speech imagined sounds that may be evoked in a passage (Brunyé et al., 2010). There is even some evidence that readers may give different characters different voices too (Alexander and Nygaard, 2008; Kurby et al., 2009). Mental imagery may also be triggered in other sensory modalities while reading like olfactory and somatosensory mental imagery. When reading olfactory descriptors, or words related to a distinctive smell, such as the word cinnamon’, has been shown to give rise to increased activation in olfactory brain areas (González et al., 2006). What is more, to the extent that the digital reading enhancing multisensory aspects of reading might provide opportunity to engage the reader’s mental imagery capacities so fully. Crucially, multisensory presentation in evoked mental imagery supports readers in the learning process, from language comprehension (Bottini et al., 1994), to socially-motivated behaviors such as perspective taking (Decety and Ruby, 2001), to motor learning (Yágüez et al., 1998). Researchers have reported multisensory approach was not only effective for students with dyslexia or reading difficulties who are learning to read and write (Warnick and Caldarella, 2016; Soliman and Al-Madani, 2017; Gori et al., 2020), but could also facilitate student learning outcomes, such as the integration between vocal signals and visual cues (like facial movements) in social and language learning (King and Lewkowicz, 2012; Weatherhead and White, 2017), the association of spoken words and visually presented objects in the context of vocabulary learning (Pereira et al., 2014), and the acquisition of speech sound-letter correspondences during reading development (Horbach et al., 2015). In recent years, multisensory experience was also designed for library reading promotion, book exhibition, music reading and art appreciation (Spence, 2020; Arora, 2021; Paraskevopoulos et al., 2021).

Recognizing the importance of multisensory attributes, institutions have begun to organize events. In 2013, the Canadian Library participated in a reading promotion event known as “Words on the Street,” which featured book exhibitions, handicrafts, painting, role playing and other multisensory activities and attracted the attendance and participation of 270,000 people (Yan, 2016). In Oxford’s Bodleian Library (2022) conservators often note that something from the Duke Humfrey’s Library has been brought in, based on nothing more than the smell that pervades the air when an item from the historic old reading room arrives. In order to help people cope with pandemic lockdown, the Bodleian Library released a stream of sounds normally heard in university libraries such as creaks, rustling, coughs and traffic noises, even including a recording from the Duke Humfrey’s Library (Kidd, 2020). Recognizing the importance of multisensory attributes, sensory events have been organized at St. Paul’s Cathedral Library (Bembibre and Strlič, 2017), the Birmingham Museum and Art Gallery, and, in late 2020, the book exhibition at the Weston Library, part of Oxford’s Bodleian Library (De Bruxelles, 2020). In China, “Modeling Library” activities in Xiamen Library in 2020 provided multisensory experiences, which made children’s reading more vivid and interesting (Song, 2022). By analyzing activities at the “Chinese Library Reading Club,” Zhongshan Library of Guangdong Province in 2018 concluded that public libraries also needed to conduct multisensory reading promotional activities for minors to effectively promote the quality and efficiency of services (Song, 2022). For academic libraries, multisensory reading promotion has been proposed as an innovative form suitable to various reader groups (Zhang and Zang, 2018). The library of Zhengzhou University in 2021 set up the “Book Sounds in Zhengzhou University” column by collecting students’ audio literary works read aloud by students, accompanied by the text and illustrations of the books, allowing readers to experience the mood and connotation of the literary works from both the visual and auditory senses (Song, 2022). Liu designed a multisensory model for reading promotion in academic libraries in 2019, showing that the mode could motivate college students’ reading, provide a multisensory reading experience, and ultimately achieve the purpose of independent and deep reading (Liu, 2019).

Many scholars have come to acknowledge that multisensory presentation [for instance, how a curator might attempt to recreate the sights, sounds, and smells associated with the Canterbury Tales without it necessarily relating to the book itself (Aggleton and Waskett, 1999)] is an innovative and effective mode for promoting reading in libraries. Yet, based on the above literature, there is little research literature on multisensory reading promotion in academic libraries and long-term extensive research is insufficient. By identifying the research gaps, we derive the following research questions to be explored: how can multisensory presentations be integrated into library reading promotional activities, and how effective is multisensory reading promotion in improving utilization of library collections? Matusz et al., (2019) pointed out that multisensory contexts reflecting naturalistic settings, provide an adaptive benefit for learning. Logically, the gain afforded by multisensory presentation can improve higher-level reading trends such as the number of reading interests, level of enjoyment, engagement etc. Therefore, we conducted an experimental study for more than 1 year on multisensory mode reading promotion implemented in the RTLE program. We expect the study results could answer the above research questions and provide a reference for other libraries.

## Methodology

Our research used the case study approach to evaluate the effectiveness of the RTLE reading promotion program, which is carried out on a library WeChat public account. WeChat is the most popular mobile application among college students and has already been employed in 84.6% of Chinese well-known university libraries (Wei and Yang, 2017). The project involved 48 librarians and 105 students who were recruited into different groups to co-create, edit, and release tweets every workday. To achieve eye-catching, ear-occupying, and heart-touching effects, RTLE presents multisensory content and encourages readers’ interactions on the platform to evoke their visual, audio, and emotional senses. RTLE effectiveness in reading promotion was also determined using the Context, Input, Process and Product (CIPP) evaluation method to measure context, input, process, and results. In the section that follows, we take a closer look at multisensory presentation in the RTLE program and the effectiveness of multisensory reading promotion on the utilization of library collections.

### Multisensory reading contents

#### Text-based content

Traditionally, librarians or students organize and participate in text writing unilaterally. This is in contrast with the RDLE program, whose contents are jointly created by them. All librarians participate in creating essays to highlight the contents of good books, while the written part of tweet could also be produced by the excellent students recruited from activities of the award solicit article. To increase reading interest and attention, the text part of each tweet is blended with theme-relevant pictures, characters’ portraits, and a story background introduction. Before their release, the program committee carries out a final polishing of the texts to match them with the selected theme. Cover pictures, library codes, and library location are included in each tweet, so that students can promptly find the recommended books.

#### Audio-based content

The program, committed to transmitting the beauty of audio reading and enhancing communication with readers, has invited students majoring or interested in broadcasting to obtain sound recordings. Approximately 3–5 min of audio frequency as reading guidance were generated by students from the excerpts of the recommended books. The anchor’s introduction is also present at the beginning of each tweet to encourage other students to take part in the broadcasting work. By listening to the classic excerpts, readers vividly comprehend the spirit of the book, and its breadth and depth can be extended.

#### Emotional resonance

It is difficult to achieve an emotional relationship with a text in the absence of participation. In RTLE, librarians’ and students’ engagement generates both service and products such as text and audio frequency. They make use of their own knowledge and give full play to their own expertise on understanding the recommended books in deeply collation and refinement from a unique personal perspective. Relying on the social attributes of the WeChat platform, readers’ feedback and comments on contents or program have been encouraged and collected. In that way, readers’ sympathetic responses echo to the mood that refers to a state of creators’ understanding of the objective.

### Effectiveness of multisensory reading

Based on the CIPP evaluation method, originally proposed by L.D. Stufflebeam, a famous American evaluation expert (Lv, 2021), the effectiveness of RTLE multisensory reading promotion is analyzed to optimize services through a continuous “evaluation-feedback” process. Context evaluation focuses on readers’ needs, existing problems, and opportunities before the program is conducted. Input evaluation, based on the context evaluation, analyzes the required resources, conditions, and feasibility of the program. Process evaluation collects data and provides feedback throughout program implementation. Result evaluation makes use of quantitative or qualitative methods to examine the degree of accomplishment of the expected objectives.

According to the CIPP evaluation method together with the RTLE reading promotion process, the evaluation steps were as follows: Context evaluation considered readers’ multisensory and fragmented reading needs and the dilemma of the continuous decline of book lending in academic libraries (mentioned in the Introduction). Input evaluation analyzed human resources, the WeChat platform, the number and types of recommended books and the multisensory presentation involving original text, audio frequency and pictures. Process evaluation collected page views and re-postings of tweets along with readers’ feedback. Result evaluation calculated the borrowing volume of recommended books. Since its launch, the RTLE program has made great strides and established a good reputation, capturing positive comments from readers. The quantitative and qualitative evaluations of the RTLE program are analyzed in the sections that follow.

#### Reading today listening everyday reading promotion data

The achievements of RTLE of multisensory reading promotion throughout 2020 are described in Table 1. All its tweets have been classified by theme into four categories: literary, cultural, historical, and comprehensive. Comprehensive themes include technology, stories of role models, and anti-coronavirus knowledge. The number and page views of different themes are shown in Table 2.

**Table 1:** The achievements of RTLE program.

Issues	Audio frequencies	Recommended books	Participating librarians	Student anchors	Page views	Re-posting
269	269	484	48	52	80,268	2,108

**Table 2:** Number and page views of different themes.

Theme	Number of tweets	Page views	Average page views of each tweet
Comprehensiveness	62	19,925	321.37
Literature	74	22,216	300.21
Culture	65	18,860	290.15
History	68	19,267	283.34
Total	269	80,268	298.39

One tweet per weekday is released on the library public WeChat account of Zhejiang University of Technology, and the weekend release is added when it comes to special days such as traditional festivals, anniversary of famous people etc. In 2020, 269 issues were released, with 80,268 page views. Page view growth is largely a function of the appeal of the content of RDLE program, which depends on the caliber of the reading promoter behind it. In RTLE, 52 student anchors and 48 librarians of different backgrounds have exploited their advantages so that every issue was told vividly and freshly from a particular viewpoint. Students participated in recording 269 audios and interacted with their comments, while librarians wrote over 800,000 words of original text and recommended 484 classic books for students. Although students are easily attracted by “fast–food type” entertainment culture, we are seeing more willingness to participate and interact in the program, with 2,108 active re-postings.

As shown in Table 2, page views varied by theme. Consistent with the statistical results, we found that students were most interested in the themes of technology, role models, and anti-coronavirus knowledge, with an average of 321.37 page views. The factor of psychological needs could be, at least in part, at play. The coronavirus pandemic, initiated at the start of 2020, aroused students’ anxiety and stress due to uncertainties in its control. The multisensory mode reading promotion attracts students’ attention and the gain of reading from relative scientific approaches, anti-coronavirus knowledge and books helps build up students’ confidence to fight against the pandemic, so that the sort of theme was most welcomed. Additionally, another highly performing theme was the literary one, with 300.21 average page views, which conveyed the spirit of classic works such as poems, prose, and novels. Tweets with cultural and historical themes, respectively earning 290.15 and 283.34 average page views, have appealed well to students too.

The daily tweets were previously described in an institutional blog created by the library on the Colmenarejo campus of University Carlos III of Madrid with the goal of promoting reading among the university population (Hernandez and de la Cruz, 2007). At present, most academic libraries measure the effectiveness of reading promotion activities by counting the number of participants, the scale of activities, the number of books borrowed during and after the activities, and readers’ feedback (Lan, 2021). In 2020, 78 book-recommendation tweets were released on the library WeChat public account of Shenzhen University of Technology, with page views of 11,325 in total and 145 on average (Lv, 2021). Ma has investigated six Fine Arts Colleges and found that the book-recommendation tweets on their library WeChat public accounts have been read 6,312 times in total and 117.22 on average (Ma, 2018). By pouring more input into this multisensory and interesting mode, the RTLE program has made great strides in terms of increasing the number of page views and tweets.

#### Data of borrowing volume

To understand the relationship between tweet views and the borrowing rate of the recommended books, page views of 269 tweets were divided into 4 ranks based on their number of views: more than 1,000 which is denoted by 1,000↑, 500 -1,000, 300 -500, and less than 300 which is denoted by ↓300. Furthermore, the borrowing rates in 2020 and 2019 were compared and analyzed to examine the effect of multisensory reading promotion.

Although the borrowing rate of paper books has been significantly reduced in current times, the borrowing rate of RTLE–recommended books has increased surprisingly. As shown in Table 3, the books recommended in tweets with more than 1,000 and 500–1,000 page views have been borrowed 19 and 27 times, respectively. Both of them are over 1.69 times higher than those of the year before. The 145 books recommended in tweets with 300–500 page views also had a good performance, being borrowed 34 times, which is 1.48 times higher than their borrowing rate in the previous year. The 181 books recommended in tweets with less than 300 page views were borrowed 33 times, which is just a bit higher than their borrowing rate in the previous year. Overall, the 484 recommended books were borrowed 113 times in 2020, which is 1.46 times higher than this value in 2019 (77 times). Although the sample size of this study is small and limited to one library, we also can get some implications. Based on statistical results, more page views indicate more reader interest leading to increased borrowing. Reading in a multisensory mode is helpful for book borrowing, especially in terms of recommended books with higher page views.

**Table 3:** The borrowing volume of the recommended books.

Page views	Number of the recommended books	The borrowing volume of the recommended books in 2020	The borrowing volume of the recommended books in 2019	Borrowing rate after and before recommendation
1,000↑	61	19	11	173%
500–1,000	97	27	16	169%
300–500	145	34	23	148%
↓300	181	33	27	122%
Total	484	113	77	146%

Book recommendations in the RTLE program continued with the launch of the “RTLE @Readers’ Talk” column in 2021. In 2020, the pictorial and audio story was used as a core reading guideline to recommend books in the “RTLE @ Librarians’ Writing” column, while in 2021, interpretation of the recommended books was regarded as the key point to encourage reader feedback in the “RTLE @ Readers’ Talk” column. Using the recommendation list as the outline and book interpretation as the core, “RTLE @ Readers’ Talk” initiated teachers and students on writing book reviews. Using the RIA note reading method (original reading + concise restatement + relative application), readers concentrated and simplified the content, extracted its essence, and wrote tweets to publish on the library WeChat public account for every issue. As of July 31, 2021, 140 original audio book reviews have been published by readers. Meanwhile, in June 2021, based on a new weekly book bulletin by Yun Reading Platform, “RTLE @ To Know New Books” column, accompanied by excellent audio fragments, was launched to recommend 4–5 selected new books to readers.

In addition, the library set up customized bookshelves to display RTLE-recommended books. These RTLE shelves, different from the traditional fixed and somber counterparts, capture readers’ attention by their various shapes and vibrant red, bright yellow and ocean blue colors. The recommended books have also been put on shelves and exhibited regularly by librarians. Meanwhile, the borrowed books have been counted to analyze the effects of reading promotion. For instance, not included in Tables 3, 10 of 17 RTLE-recommended books were borrowed during the 2020 Reading Festival after 2 weeks of first-time exhibition on bookshelves. Their borrowing rate surprisingly reached 58.8%. This was not a singular case: other libraries also reported enhanced lending rate after promotional reading activities. For instance, after holding the book reading and sharing club “Reading Joy,” the relative lending rate of three recommended books at the Shenzhen University of Technology Library increased 184, 200 and 133%, respectively (Lv, 2021). Lin used the “360°-pleasant reading” carried out by the Hainan Medical College Library as a practical example; through in-depth guided reading, the lending rate of recommended books increased by 177.4, 189.2, and 170.8%, respectively (Lin, 2015). By highlighting recommendations and using a centralized display, several inactive books in the library have been reintroduced into circulation.

#### Readers’ feedback

Since its launch, RTLE has attracted the attention of more than 40,000 readers who often leave positive messages in the comment area to express their satisfaction. According to messages in the comment area, reader feedback can be summarized into the categories shown in Table 4.

**Table 4:** Readers’ feedback.

	Category	Messages
1	Reading desire has been stimulated.	*“I wish to read this book and miss the library.”* *“That play had been recommended and I want to watch it.”* *“Thanks the author to let us understand the history, a sad story, a grateful poem*!” *“There are a bunch of good anti-epidemic books waiting for me to read...”* *“Well done! Understanding the ancestors sacrificing themselves for our happiness today, we should be satisfied, grateful, learn their spirit for the revolution.”* *“I learned that, thank you!”*
2	Readers were attracted by audio frequency, picture and text.	*“I come for the cover*,” “*the audio reading is excellent, as if we are brought to the fish world.”* *“Beautiful, beautiful pictures and texts.”* *“The anchor’s audio frequency is so great that It‘s like being there.”* *“It is not easy to find an anchor who can recite the complicate book. The anchor is awesome.”* *“I wonder what is BGM，and I read it at one sitting.”* *“The anchor is very professional with solid broadcasting skills.”* *“The anchor’s voice has been echoing in my ears.”*
3	Some readers left messages to express their love for the program.	*“RTLE leads us to start a cultural journey at home.”* *“RTLE is my favorite program and I will continue reading all long.”* *“Clear context, detailed content, this typesetting is really diligently.”* *“The author writes so well.”* *“The topic is very nostalgic to make me recall the story-telling scene when I was a child.”*
4	There are more expectations.	*“RTLE as a whole should be more in-depth, not only in the aspect of book selection, but also the understanding of text and the reasons for recommendation.”* *“I hope the name of the background music would be attached in the following program.”* *“I always feel that the speed of the speech is a little bit fast.”* *“The pronunciation is good, while the intonation lag behind.”*

From the readers’ feedback, RTLE generally impressed readers. Readers’ messages expressed their love for the column in terms of audio frequency, content, pictures, typesetting, etc., their wishes to read and acquire knowledge, their recommendations, and their expectations for a better column. The comment area of high-view tweets often becomes a place for communication and interaction between library and readers, and among readers themselves. For example, one issue of “Ugly or not is unimportant, whether I have personality matters!” which narrated Zhu Yuanzhang’s magnificent entrepreneurial history, providing an image of grass-roots entrepreneurs, attracted the attention of many Ming Dynasty fans. Readers expressed their love and respect for the Ming Dynasty through their praise, left in messages on the platform. In addition, readers also recommended the classic book “Wanli Fifteen Years” of Ming history in the comment area, which provided reading guidance for other readers as well as a reference for library procurement. School alumni also left messages, “*Readers graduated several years have even been reluctant to lose access to the library’s public WeChat account.*” It can be seen from readers’ positive feedback that the multisensory mode of reading promotion could attract reader attention and lead to additional reading. In the era of new media, readers are not only content readers, but also content creators. Readers’ feedback is RTLE’s greatest driving force for continuous growth and development, so librarians set up and published various comments for readers to discuss further to evoke their reading consciousness and promote deeper reading.

### Analysis of multisensory reading

In the RTLE program, visual, audio, and emotional senses are motivated. How they work in the reading process have been analyzed in the following.

#### Analysis of visual reading

Rich visual input underlies reactivation of visual representation–that is, visual imagery–to generate reading comprehension. Importantly, the visual imagery evoked by the pictures and words is likely to set expectations in the reader’s mind regarding the nature of the contents. For example, in the background of beautiful pictures of natural scenes, the 105^th^ tweet introduced a picturesque place (Kekexili), successfully earning 523 page reviews. During the reading, visual imagery is also elicited. That is one might be filled with an awe for nature and want to consider the sensation transference that may occur, so that one expects what the contents of recommended book and the feeling of a real trip are.

#### Analysis of audio reading

Sound conveys the dynamics of the surrounding environment and provides information about its spatial, contextual, and high–level attributes to enhance the vividness of auditory imagery. The audio component of the program allowed readers to experience sound–induced cognitive processes and manipulate the interaction between perceptual and contextual cues. On May 12^th^, the tweet of “Big love follows disaster, spring will eventually come” successfully earned 2048 page views, which described an event that Wenchuan earthquake in Sichuan province happened on the same day in 2008 and the huge impacts and rapid reconstruction of the area. As the master anchor’s sound and music build to a crashing crescendo, students can be easy to identify with the mood of the coming spring, after having suffered so much during the coronavirus pandemic and having been gradually recovering from it. Arguably, the program has spiritual value, allowing students to isolate themselves from disturbances and immerse themselves in the beauty of reading and relaxing their body and mind accompanying the broadcaster’s soft voice.

#### Analysis of emotional interactions

Multisensory reading allows us to mobilize the audiences’ perceptual senses, which is the simplest and most preliminary idea regarding it. Additionally, it needs to consider strategic research, communication and interaction in activities. In RTLE, readers share their inner feelings to respond to creators’ understanding and mood to induce emotional resonance. For instance, one alumnus left the message “*On campus, it is so quiet that I can hear my heartbeats and breathing sounds, feel birds flying and hares getting through the bamboo forest as well. It is the first time I feel that the mountain is more secluded while the birds are singing.”* Mental imagery triggered by multisensory inputs may play an important role in aiding the recall of the reader’s experience and account for the program’s emotional appeal.

### Design and implementation of the RTLE program

**Figure 1:**
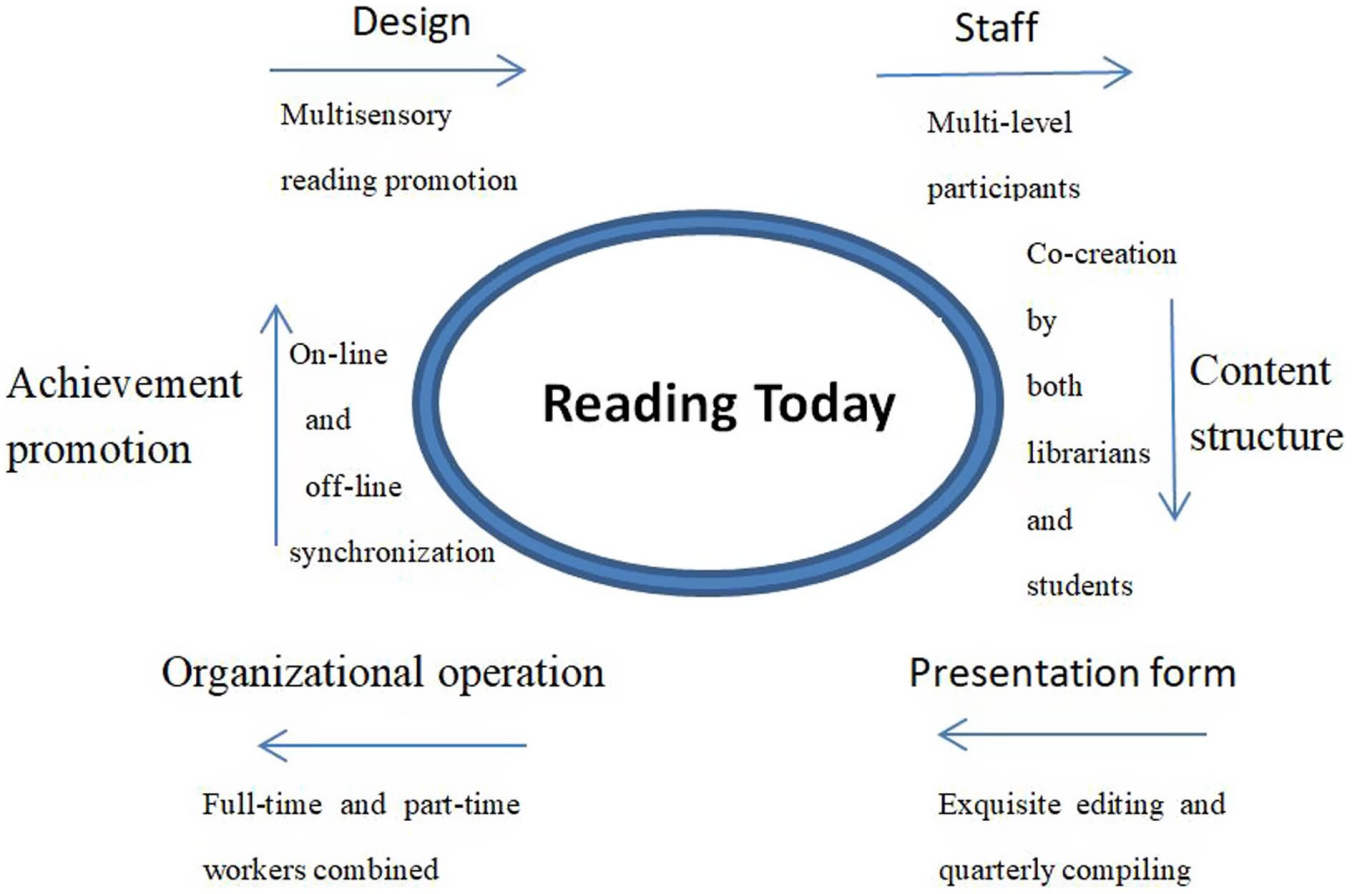
The design of RTLE program.

From 2020 to 2021, the Zhejiang University of Technology Library launched its RTLE program on the library’s WeChat public account, divided into two parts: the “RTLE @ Librarians’ writing” column in 2020 and the “RTLE @ Readers’ Talk” counterpart in 2021. Aiming to recommend library collections to guide in-depth reading, the RTLE program constructs a promotional platform featuring multisensory promotion, multi-subject participation, and multichannel dissemination. Multilevel readers are recruited into RTLE working groups and divided into full-and part-time staff. Among these, librarians and students co-create the multisensory tweet contents and jointly run the program. After meticulous editing, while the tweets are released online, the quarterly audio comic books, compiled from the contents of tweets, are disseminated offline. The process of RTLE design is illustrated in Figure 1 below.

#### Design–multisensory reading promotion

After accumulating a certain level of experience, library administrators decided to pour more inputs into this multisensory, interesting, interactive, and effective mode of reading promotion, going from producing one issue per week to producing one issue per day. RTLE advocates reading not just by seeing, but also by listening and through other multisensory ways. The program suggests an “audio plus pictorial” form of multisensory reading would provide readers with a better understanding of books. Relying on the social attributes of the WeChat platform, readers can naturally empathize with the spirit of works through their comments, messages, and discussions in comment areas to promote deeper reading. A complete closed loop of reading promotion would be created by librarians’ recommendations of books and readers’ comments on them. In a series of aural and pictorial tweets of RTLE, the authors recommend books through storytelling in an atmosphere rendered by music, human voices, pictures, and text elements.

#### Staff–multilevel participants

Characterized by flexibility and openness, RTLE is open to, and welcomes, anyone interested in the program at any time. In addition, program participants, according to their own advantages or wishes, can flexibly join one or more groups such as a “content-writing group,” an “anchor group,” an “editing group” and a “publishing group.” Program staff is composed of the library’s culture and broadcasting department, which is the full-time project component, managing program operation and personnel training; skilled and experienced librarians with excellent writing skills, recruited to ensure the quantity and quality of manuscript sources; winners of the prophase composition–writing or read-aloud contests enlisted on the team; teachers from the Humanities College invited to participate in program instruction and evaluation; broadcasting majors recommended by their professors to take on the post to gain experience; and students of the library association in charge of the WeChat editorial work. Among other things, ardent alumni and their children and friends have supported us by providing their read-aloud recordings and sharing their reading comprehension.

#### Content structure–co-creation by librarians and students

Cooperation between librarians and students enables the program to work steadily. The content structure includes text-based and audio-based portions, co-created by librarians and students, in the form of a combination of written and spoken words. In the text-based portion, most of texts were created by librarians, together with partial texts produced by outstanding students. Both librarians and students formed the “content-writing group,” the authors of which have various styles and wide interests to recommend library collections from different viewpoints. In the audio-based portion, students majoring or interested in broadcasting have been invited to make up the “anchor group” to complete sound recordings upon librarians’ training and text interpretation. Finally, the contents will be polished and verified several times by members of the “editing group” and the “publishing group” to ensure the release of high-value essays. RTLE has absorbed both the reader group and the librarian group to form a major alliance of user-produced content represented by student readers and professional content producers represented by librarians.

#### Presentation form–meticulous editing and quarterly compiling

Following the completion of text and audio frequency, each tweet is rolled out after it is edited by work-group members, combining it with relevant pictures and information to increase reading interest and attention. Audio and video resources released on the library’s public account have been stored on the WeChat platform for reader access at any time. Additionally, all materials are compiled in every quarter, including a quarterly disk of audio frequency and print booklet of text contents, which are preserved as references for subsequent activities. Although audio disks have not yet been collected in the library, they can be lent to support use when books are introduced inside and outside libraries. In addition, the library has also successively printed RTLE content into audio comic books as a supplementary material for offline reading guidance.

#### Organizational operation: A combination of full- and part-time workers

Program implementation and management are carried out jointly by librarians, students, teachers, alumni, etc. In terms of personnel arrangements, the RTLE program has set up the full-time three-librarian Culture and Broadcasting Department and recruited 165 part-time reading promoters. The three full-time librarians are responsible for project planning, preparation, implementation, evaluation, and coordination and management of all part-time staff. The part-time team is composed of a variety of members, including 105 part-time student promoters (52 student anchors and 53 student volunteers), 48 librarians from other departments, and a total of 12 outside reading promoters (including two librarian friends, three children of alumni, one broadcaster from China Central Television, one chairman of the Disabled Persons’ Federation, one retired teacher, and four rural commissioners). All part-time student promoters were grouped into shifts, with each group containing a leader (who could be in three to five groups), a typesetter, a copywriter and a photo editor.

#### Achievement promotion–on-line and off-line synchronization

The RTLE reading promotion program features integration of online micro-reading and offline deep reading. During the continuous push of the online WeChat column, relevant reading activities surrounding RTLE themes were regularly carried out, including extended online activities such as the classic read-aloud activity “Hearing Your Voice,” the broadcasting drama “Long Word,” the activity of new book recommendation “Getting to Know New Books,” and supporting offline activities like reading conferences, book fairs, related lectures and essay contests, etc. In addition, the RTLE materials have been rearranged, compiled, and printed into a total of 1,200 audio comic books, divided quarterly (spring, summer, autumn and winter) to be donated and distributed to eight rural cooperative grid reading rooms to enrich the local reading room collection and help create a positive reading atmosphere.

## Implications

Summarizing the relative activity experiences can help innovate mode of library reading promotion and explore much higher and deeper level of reader service. The RTLE program provides a multisensory reading experience by tapping into the potential of students’ visual and audio abilities with pictorial and aural stories and essays. Moreover, the further interaction of commenters allows for emotional resonance and guides students to switch from shallow reading to deep reading. This enlightening and thought-provoking multisensory reading is not only considered as a new mode of reading promotion but also as an innovative academic library service. As mentioned in the previous part of the article, other libraries should give attention to the multisensory turn. For instance, Bembibre and Strlič (2017) analyzed samples from an old book which develop a ‘historic book odour wheel’. And, as for many other odour/flavour wheels, the idea here is that this helps connect identifiable chemicals with people’s reactions to them (Bembibre and Strlič, 2017; Spence, 2020). Beyond the sound of the books themselves as they are interacted with, there is also the unique sound scope of the library. For example, in Duke Humfrey’s library, a stream of the sounds of creaks, rustles, coughs, and traffic noises that you could normally hear could be released to help cope with the pandemic lockdown (Kidd, 2020). A growing recognition of the importance of the multisensory attributes of reading has led a number of libraries, museums, and archives to organize exhibitions that engage more visitor senses (Classen, 2016).

In the social background that sees the government actively promoting nationwide reading, the system-mature, widely point-distributed, and resource-rich public interest institution of the library has naturally become the core force of reading promotion. Its excellent cases and successful practices are certain to provide references for social reading (Li et al., 2020). In the present study, we found that multisensory reading attracted readers’ attention, stimulated their reading interests, and promoted their reading practice. As the continuous development of social reading has spawned new reader groups and new reading service needs, multisensory participation in reading may provide a possibility to meet their needs with the assistance of modern technology. For instance, even if you do not sit down and read quietly, you can listen to the indirect or wonderful fragments of a book in a busy environment. Thus, integrating reading into a multisensory context can be extended to social reading to promote nationwide reading and construct a scholarly society, further enriching and expanding the theory of reading promotion and providing advice for administrative policy making. Similarly, a multisensory approach could also be of pedagogical benefits to arouse students’ attention, increase learners’ engagement in activities, and improve students’ academic outcomes. In pandemic times, the attention-arousing and interest-stimulating mode of multisensory reading in RTLE delivers a value of book with words and sound that can soothe and heal fear, anxiety, and other stressful emotions and solve the dilemma of college students’ lack of spiritual guidance. Moreover, the present study hopes to help academic libraries to play their role in public emergencies.

It is important to mention some limitations of the present study. First, what we concluded from the present study based on the data of multisensory reading carried out in the RTLE program, in which readers are restricted to college students and the samples are small. Multisensory contexts are natural for life-long learning, so further research on big-sample and multi-level learners like schoolchildren, preschoolers, infants, and elderly people is necessary. Second, the multisensory presence of our study appears in the form of visual, audio, and emotional senses, which is somewhat different from a unisensory presentation. However, the addition of other perceptual modalities is supposed to flourish multisensory capabilities in future research, a relatively understudied topic. Third, the present study detected the effectiveness of multisensory stimuli in library reading promotion but did not observe differences between specific deviants like visual, audio, and emotional senses and evidence for implicit cross-modal correspondences. Nonetheless, it would be informative for future research to determine what might constitute an optional approach in terms of reading promotion in public or academic libraries and educational institutions.

## Conclusion

The present study on reliable links between multisensory modes of reading promotion and students’ reading tendencies cannot directly speak to their causality. Nonetheless, our results on the RTLE program would indeed suggest that multisensory inputs constitute an effective access to promote students’ reading practice. They also support the applicability of multisensory modes of reading promotion to innovate library services. Although multisensory reading in the RTLE program remains a trial limited to visual, auditory, and emotional senses, its functions are certain to undergo further developments, along with the popularity of the program, the increasing growth of the WeChat platform, and the possibility of using other new media technology and the addition of other perceptual modalities. It would be particularly promising to apply multisensory reading promotion in other libraries, such as developing a new extension service and integrating multisensory engagement in social reading. Moreover, combined with a prompt administration time, the multisensory method can be an attractive potential didactic tool for student learning.

## Data availability statement

The original contributions presented in the study are included in the article/supplementary material, further inquiries can be directed to the corresponding authors.

## Author contributions

YW: conception and design of the study, and writing—original draft. YJ: data curation and resource. WY: analysis of data and validation. YC: investigation and supervision. All authors contributed to the article and approved the submitted version.

## Funding

This research was funded by National Social Science Foundation of China (20BTQ028), Humanities and Social Sciences Research Fund of the Chinese Ministry of Education (22YJC870010), and the Philosophy and Social Science Fund of Zhejiang Province (21NDJC039YB).

## Conflict of interest

The authors declare that the research was conducted in the absence of any commercial or financial relationships that could be construed as a potential conflict of interest.

## Publisher’s note

All claims expressed in this article are solely those of the authors and do not necessarily represent those of their affiliated organizations, or those of the publisher, the editors and the reviewers. Any product that may be evaluated in this article, or claim that may be made by its manufacturer, is not guaranteed or endorsed by the publisher.
